# The embodied transcendental: a Kantian perspective on neurophenomenology

**DOI:** 10.3389/fnhum.2013.00611

**Published:** 2013-09-30

**Authors:** Omar T. Khachouf, Stefano Poletti, Giuseppe Pagnoni

**Affiliations:** ^1^Department of Neural, Biomedical, and Metabolic Sciences, University of Modena and Reggio EmiliaModena, Italy; ^2^Department of Applied Cognitive Psychology, University of BolognaBologna, Italy

**Keywords:** neurophenomenology, Kant, a priori, prereflective awareness, default mode network, ongoing activity, free-energy, meditation

## Abstract

Neurophenomenology is a research programme aimed at bridging the explanatory gap between first-person subjective experience and neurophysiological third-person data, through an embodied and enactive approach to the biology of consciousness. The present proposal attempts to further characterize the bodily basis of the mind by adopting a naturalistic view of the phenomenological concept of intentionality as the *a priori* invariant character of any lived experience. Building on the Kantian definition of transcendentality as “what concerns the *a priori* formal structures of the subject's mind” and as a precondition for the very possibility of human knowledge, we will suggest that this transcendental core may in fact be rooted in biology and can be examined within an extension of the theory of autopoiesis. The argument will be first clarified by examining its application to previously proposed elementary autopoietic models, to the bacterium, and to the immune system; it will be then further substantiated and illustrated by examining the mirror-neuron system and the default mode network as biological instances exemplifying the enactive nature of knowledge, and by discussing the phenomenological aspects of selected neurological conditions (neglect, schizophrenia). In this context, the free-energy principle proposed recently by Karl Friston will be briefly introduced as a rigorous, neurally-plausible framework that seems to accomodate optimally these ideas. While our approach is biologically-inspired, we will maintain that lived first-person experience is still critical for a better understanding of brain function, based on our argument that the former and the latter share the same transcendental structure. Finally, the role that disciplined contemplative practices can play to this aim, and an interpretation of the cognitive processes taking place during meditation under this perspective, will be also discussed.

## Introduction

Neurophenomenology, a programmatic endeavour to integrate the basic principles of Edmund Husserl's phenomenology with the findings of cognitive neuroscience, was originally proposed within the theoretical framework of autopoiesis and enactive cognition (Maturana and Varela, 1980; Varela et al., 1991) as “a methodological remedy for the hard problem” (Varela, 1996). While its aim of bridging the explanatory gap between first-person witnessing of life and third-person scientific accounts of experience has yet to be fulfilled, there is currently a renewed interest not only in better defining the theoretical project itself, but also in identifying a pragmatic implementation of the neurophenomenological method (Lutz, 2002; Lutz et al., 2002; Lutz and Thompson, 2003; Cosmelli et al., 2004; Thompson, 2007).

Within the dialectic field spanned by the first- and third-person epistemological poles, many phenomenology-oriented authors have recently argued for at least a methodological, if not an ontological primacy of consciousness over its neuroscientific correlates (Wallace, 2000; Bitbol, 2008). Bitbol (2008), in particular, uses a number of arguments from epistemology, phenomenology, neuropsychology, and physics to demonstrate the inconsistency of a reductionist approach to consciousness, where mental states are ontologically dependent on physical states; neurophenomenology is then viewed as a novel scientific method building on a corpus of intersubjectively-invariant first-person reports that may broaden the horizon of objective science.

In this paper, we would like to take a closer look at and build on one of the key features of the enactive approach, namely the natural roots of intentionality, a phenomenological notion indicating that experience is always “about something” (Thompson, 2007, p. 27, pp. 157–162). To this aim, we will argue that for the environment to become *meaningful* for an organism, the latter must be endowed with a hierarchical set of *a priori* (albeit malleable) structures that somehow mirror selected aspects of it—in line with Kant's notion of transcendental. Since the transcendental is also at the very basis of phenomenology, we hope that underscoring its embodied roots can provide a useful inspiration for future interdisciplinary research into the mind-brain problem.

**Figure 1:**
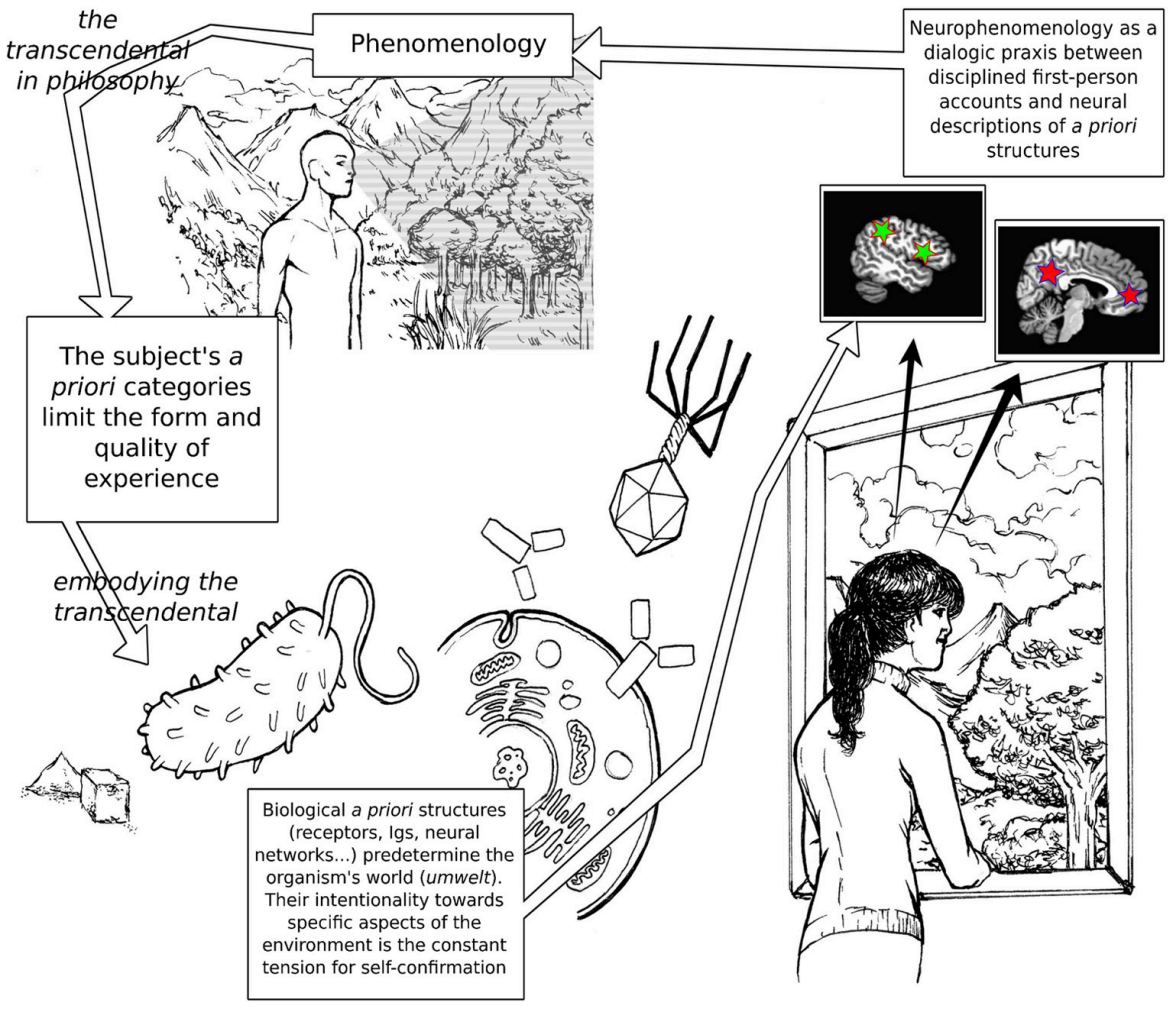
**A schematic depiction of the articulation of the main themes of the article**. The brain pictures are toy-representations of the default mode network (red stars) and the putative human analog of the mirror-neuron circuit (green stars). On the lower left of the figure, a flagellated bacterium is moving toward an area of high sucrose concentration. In the middle, Y-shaped immunoglobulines are poised to recognize a viral epitope.

This article is structured as follows. After introducing the philosophical background to our thesis (Section 1), we will discuss a naturalized account of intentionality, whereby the transcendental is interpreted as the defining character of autopoietic agents (Section 2); the bacterium and the immune system will be used as elementary examples of embodied transcendentality. In Section 3, we will propose that the activity of selected neural networks in the human brain can be interpreted as displaying the functionality of the transcendental structure at multiple levels, suggesting potential implications for clinical conditions; the free-energy principle proposed by Karl Friston (Friston and Stephan, 2007) will be introduced as a neurobiologically-plausible theoretical framework that seems particularly fitting for the ideas presented here. We will conclude (Section 4) with some considerations on the role that contemplative practices may play in neurophenomenology. Figure 1 illustrates synoptically the relationships among the themes discussed in the present paper.

## 1. The transcendental in philosophy

We will begin by introducing a few fundamental concepts from Husserl's philosophy (Husserl, 1960, 1970), with an emphasis on their Kantian roots, in order to characterize the role of the transcendental in our own proposal.

### Husserl's transcendental phenomenology

Husserl refuses to accept what he calls “the natural attitude,” the naive and non-reflexive everyday consciousness of the world leading to the common belief that reality as it appears exists in itself, that is, has an ontological value. Husserl's operation, called *epoché*, consists in the attempt of “bracketing out” unexamined preconceptions from statements about reality (including those on which natural science relies), and finds the manifestation of consciousness itself as the only residual of this rigorous examination. This gnoseological *praxis* led Husserl to the characterization of subjectivity as transcendental, a term denoting the *a priori*^1^ determination of the form and quality of experience. According to Husserl, the fundamental character of experience does not consist in its phenomenal content, but rather in the pre-given “horizon” (a term with a deliberate connotation of “illusory” or “apparent”) that is the condition for the perception of each object or phenomenon: this background primary consciousness is what enables the transcendentally-constructed world.

### Intentionality and prereflective awareness

We will use the term “intentionality” here to indicate the mind's innate tension toward its object, a definition underscoring the relationship between act and content of experience. Mental acts or processes (e.g., focusing one's attention, recalling memories, experiencing surprise to unexpected events, etc.) are referred to by Husserl as *noesis*; mental contents, such as objects of perception, thought, memory, imagery, emotion and so on, are called *noema*. In order to understand the forthcoming sections, it is useful to see *noesis* as an *a priori* dimension of experience, and *noema* as an *a posteriori*, although such distinction may not correspond exactly to Husserl's original position. Moreover—since to see an object is indissolubly tied to the subjective experience of seeing, to recall a memory cannot be separated from the subjective quality of recalling, and so on—intentionality is coessential with prereflective self-awareness (Lutz and Thompson, 2003), the self-manifesting awareness of experience that does not require a voluntary act of introspection or reflection (Depraz et al., 2000; Zahavi, 2003). Prereflective self-awareness is considered by a long philosophical tradition as the very mode of experience (“any conscious existence exists *qua* conscious of existing,” Sartre, 1943; Zahavi, 2003), and there has been recently a keen interest in the search for its bodily roots (Wider, 1997; Zahavi, 2002). Notably, prereflective self-awareness can be seen as the most basic form of *noesis*, i.e., the fundamental *a priori* form within whose limits all experience arises. It is in this sense that we can view intentionality as a manifestation of the transcendental: to use a metaphor, it can be likened to the founding act of the fisherman casting his net out into the sea to begin his catch; without this initial lighting up of consciousness, which embeds an essential *predictive* component, nothing could be perceived at all.

It is important to distinguish this notion of intentionality from its functionalist-cognitivist acceptation (Fodor, 1975), the latter indicating the semantic link between a mental representation and its object in the external world that often assumes a one-to-one mapping. Concerning the validity of this assumption, it is useful to briefly recall here Freeman and Skarda (1990)'s argument about the widespread use of the notion of representation in cognitive science. In a cogent critique, the authors point to a consistent body of experimental evidence that the search for specific EEG patterns coding for different olfactory stimuli had been misleading: odor-specific neural activity in the olfactory bulb is in fact more influenced by the ongoing neural, behavioral, and environmental context than by the sheer physical characteristics of the external stimuli (Freeman and Skarda, 1985), a finding that is difficult to reconcile with a purely representationalist view of cognition.

### The *a priori* determination of knowledge: Kant's legacy

Before delving further into the matter, we would like to consider briefly the Kantian legacy in phenomenology. Kant aimed at providing a theoretical justification for the objectiveness of the newborn Galileian-Newtonian physics. The 18th century debate about the legitimacy of a mathematical formalization of nature, i.e., about whether nature could conform to a merely-human logic, led the skeptic philosophers (Hume, Berkeley) to claim that the only value that can be attributed to science is practical and nothing can be stated about its connection with reality. In order to overcome such impasse, Kant proposed a revolutionary (albeit influenced by Aristotle) reconceptualization of the process of human knowledge, which he illustrated in his *Critique of Pure Reason*.

Kant begins by examining perceptions (called “sensory intuitions”) as the result of the encounter between the external world and the subject's senses, where every perception is necessarily framed within a specific space and time. However, the categories of space and time are now considered neither absolute and objective, i.e., existing in the world (as Newton held), nor totally subjective and existentially private (as Leibnitz claimed). Space and time are for the first time defined as “transcendental,” that is, formal and empty *a priori* structures of the subject. The external world can be sensorily perceived only inasmuch as it “fits” such predetermined forms. The thus obtained sensory intuition is then passed onto a higher level of cognitive elaboration where a different and more abstract set of categories (including quantity, quality, reciprocity, cause-effect, etc.) mediates the production of scientific claims—propositions that are by definition true both *per se* and intersubjectively. But how can the link between these intellectual categories and the outer world—and therefore the objectivity of science—be guaranteed? Intellectual categories must be transcendental forms as well, which thanks to a direct connection to the empirical senses via the basic space and time categories, can predetermine the kind of external material that can be sensorily perceived. To put it succintly, we can only perceive what we can elaborate into concepts.

The *Critique of Pure Reason* indeed overturns the relationship between knowing subject and experienced object, arguing that the properties that we can assign to the object are nothing but the very preconditions for knowing the object itself: we do not know the object *per se*, and our world is populated by objects only inasmuch as they fit our predetermined sensory and intellective apparatus^2^. It is not difficult to extend such notion to living beings in general, within the perspective of enactive cognition: each organism brings forth a predetermined (but malleable) structure to face the world, thus creating its own *umwelt* or “inner world” (Uexkull, 2010). To use a common illustration, a bacterium sensible to three chemicals (e.g., sucrose, lactose and an isomer of lactose) lives in a world whose cardinal features consist only of these three objects fitting the bacterium's predetermined sensory apparatus (receptors and metabolic networks): the rest of the physicochemical world for the bacterium is the “the object *per se*,” i.e., Kant's *noumenon*, which is utterly opaque to cognition. From such a point of view, the transcendental can in fact be appreciated as the very mode of both subjectivity and life.

## 2. Embodying the transcendental

A number of different nuances about the notion of autopoiesis were highlighted already in Varela et al. (1974); Maturana and Varela (1980). A simple yet precise definition, taken from Varela's later writings and adopted by Thompson (2007) as a cornerstone for his argument, is that a system can be considered autopoietic if 1) it consists of a network of chemical reactions which regenerate at least some of the components of the system, 2) the system has a semipermeable boundary, and 3) this boundary is the result of reactions taking place necessarily within the boundary. The term “operational closure” is used in Varela et al. (1991) to indicate the intrinsically recursive nature of the reactions the system consists of. Since such a system is immersed in the environment it generated from and continually exchanges energy and matter with it, the identity singled out by the autocatalytic production^3^ of the system's membrane is far from representing a disconnection from the external world. Indeed, its identity emerges from bringing forth selected relations with the environment, such as the intake of external chemicals that will take part in the system's reactions: these chemicals become *nutrients*, acquiring that “surplus of significance” that points to the difference between external *environment* and *world* under the organism's perspective (Varela et al., 1991; Varela, 1997). Operational closure and the environment-linked thermodynamic openness of autopoietic systems can be described as a continuous change within a struggle for the re-affirmation of an invariant form. Such relationship between autopoietic agents and the environment (*enaction*) is what characterizes, in Varela and Maturana's view, the minimal form of cognition, as synthesized by the formula “living is sense-making” (Varela et al., 1991).

Our contribution to this view focuses on the examination of what enables the organism to make sense of the environment (however unconsciously). We propose that an embodied analog of Kant's *a priori* structures may be at work. This change of accent, within the same perspective, is based on an extension of the notion of the Kantian transcendental consisting in rooting an *a priori* formal structure at the biological level. We acknowledge here a strong affinity with Thompson (2004), who links intentionality as a self-organizing openness to the world with biology, arguing that autopoiesis is “the minimal form this type of self-organization can take” (Thompson, 2007, pp. 157–165). This sentence contains indeed, in a nutshell, a formulation of an embodiment of the transcendental similar to ours, with an explicit reference to dynamic systems theory (Thompson, 2007, p. 27). However, Thompson's claim that experience is “irreducible” due to its “ineliminable transcendental character”(Thompson, 2004) seems to favor a usage of the transcendental in a purely-phenomenological way: the transcendental is our own lived experience, which alone renders an epistemology of living organisms possible (Thompson, 2007, pp. 162–165). Our analysis of the autopoietic analogs of Kantian categories, on the other hand, aspires to trace the “ineliminable transcendental character” of phenomenology within biology (and the brain) itself. In order to clarify this important point, we discuss briefly some relevant issues highlighted by Thompson (2007).

### Autopoiesis, life, and cognition: one and the same?

In the original formulations (Maturana and Varela, 1980; Varela, 1997), the notions of autopoiesis, life, and cognition are characterized by a marked coextension and interdependence of their meanings. However, such equivalence—and in particular the assimilation of cognition to autopoiesis—has been recently questioned. In the following, we briefly outline the arguments of two of the main contributions to this debate [see Thompson (2007) for a review].

Bourgine and Stewart (2004) illustrate their critique by devising a mathematical model of a closed dynamical system implementing a virtual structure similar to a micelle^4^. The reactions occurring within its boundary satisfy the conditions for the system to be called autopoietic: they continuously produce their components and the boundary, which in turn encloses the reactions. A single chemical A permeates the membrane and is the substrate component of the main reaction of the system (*A* + *A* → *B*), catalyzed by the components C of the boundary. As components C decay on the boundary and are released in the milieu as components D, holes are progressively formed on the boundary's surface. These holes are repaired by components B, which are transformed into C components if they come in contact with the edge of a hole. The automaton can either maintain itself (if membrane holes are under a certain threshold, in terms of number and size), or collapse (if this threshold is exceeded), depending on the respective velocities of the two reactions of formation and decay of the components of the boundary. However, despite its autopoietic nature, no behavioral (motor) reaction is produced by the virtual micelle in response to nutrients income (interpreted as sensory data). The realization of a system with autopoietic features but devoid of a fundamental sensorimotor loop, which is deemed essential for cognition by the authors, leads the latter to refute the equivalence of autopoiesis and cognition.

A similar conclusion is achieved by Bitbol and Luisi (2004), who exemplify their point by considering a liposome, a vesicle-shaped self-organizing chemical structure whose boundary is made of a bilayer of lipidic molecules, called S (surfactant). The aqueous environment of the liposome contains a highly lipophilic precursor of S (called S-S) that is able to bind the vesicle's bilayer and, through an autocatalytic reaction of hydrolysis, integrate as S-components into the boundary, thus enlarging its surface area. After several hydrolysis reactions and integrations, the liposome eventually divides into smaller vesicles (a variant of the same experimental model characterized by *homeostatic*, rather than *self-reproducing* autopoiesis has also been devised). Bitbol and Luisi argue that while such a model qualifies as autopoietic, it does not exhibit (elementary) cognitive properties. For the authors, in keeping with Maturana and Varela's original view, the essence of cognition lies in the continuously unfolding interactions between the organism and the environment, whereby the former operates an active selection on the latter to ensure its own viability. Crucially, this process can be recast in terms of the workings of a living system's metabolic network, which mediates the choice of suitable metabolites from the environment and expels waste catabolites in it, a metabolic network that is absent by design in their example autopoietic system.

Both Bourgine and Stewart (2004) and Bitbol and Luisi (2004) mention additional conditions for an autopoietic system to qualify as a living one. Thompson (2007) suggests that the whole issue may simply stem from a choice of terms: a narrower definition of autopoiesis may not necessarily entail life and cognition, but if we adopt a broader definition that requires the workings of a complex metabolic self-organizing network, we could just consider the examples from the authors above as proto- (or pre-) autopoietic, rather than minimally-autopoietic, and leave the original equation between autopoiesis, life and cognition, intact.

We have reviewed these points with the intent of providing a context for our proposal and clarify its contribution. Quite independently of the chosen definition of autopoiesis, we believe (a) that the recognition of a fundamental similarity between mind and life represents indeed a useful starting point for both theoretical speculations and empirical research, and (b) that such similarity should be sought as far down the evolutionary ladder as an embodiment of the transcendental perspective can be traced. From this point of view, the models proposed by the aforementioned authors do not exhibit cognitive properties precisely because they lack such embodiment, which impinges on the presence of a form enabling the organism to distinguish between useful and irrelevant dimensions of the environment and is a necessary prerequisite for the kind of mental act that in Section 1 we compared to “a fisherman casting his net out into the sea”. In order for this intentionality—and the “surplus of significance” it entails—to arise, concrete structures must be already in place that select out features from the environment that are *transcendentally* meaningful for the organism, relying on a sensorium whose very existence descends from the iterated coupling (across multiple time scales) of organism and environment.

Let us consider first Bourgine and Stewart's autopoietic automaton: in the virtual micelle, a few reactions take place and produce some of their own components and those of the boundary. For these reactions to occur, a chemical coming from outside the boundary and permeating it is needed. In our perspective, such a system does not possess cognitive properties because it does not feature any explicit, appropriately-shaped organ^5^ oriented to the detection of a specific chemical. More precisely, the surface membrane of the autopoietic micelle has no selective receptors or gates for the nutrients necessary for its sustenance—receptors embodying the portions of the environment that subserve the system's “natural purpose” of continued existence (Weber and Varela, 2002) —and therefore does not entail any attempt to “get a grasp on” (a “com-prehension” of) the environment. Likewise, Bitbol and Luisi's model of minimal autopoiesis also lacks the embodied transcendentality of *a priori* structures that would enable the vesicles to select appropriate chemicals out of the environment and process them within an internal metabolic network. The precursor S-S comes in contact with the boundary of the vesicle only incidentally and reacts with it by enlarging its surface, without any autonomous tendency of the system to an openness to the external milieu.

### The diachronic and synchronic aspects of the transcendental

Before examining a selection of scientific data under the theoretical framework of embodied transcendentality, we would like to point out that the search for embodied *a priori* structures that enable cognition in an organism may be biased toward focusing on a single time-slice selected out of a continuously-occurring dialectical relationship between organism and environment. While we believe that this is a useful procedure, it is important to note that the transcendental process arguably operates at multiple temporal scales. In particular, following Thompson (2007)'s usage of concepts from developmental systems theory (Oyama, 2000a,b), the behavior of ecologically-embedded organisms can be seen as contributing to a modification of the environment itself (through the creation of niches), which can thus be considered part of the hereditary endowment of the organism. Crucially, natural selection operates on the integrated developmental system of organism *plus* environment and, in this “diachronic” perspective, evolution can be seen as the process of embodying in the organism the *a priori* structures that mediate its viability. It is because of their emanation from the coupling history of organism and environment that such structures enable a formal “resonance” between the former and the latter, which represents the “synchronic” aspect of transcendentality underlying the creation of a meaningful world for the organism. We anticipate that the distinction between the synchronic and diachronic aspects of transcendentality is echoed in virtually identical terms by the distinction between the processes that give rise to perception and those that change the architecture of the model of the world embodied by the organism in terms of minimization of free-energy (Friston and Stephan, 2007; Friston, 2010a) (see Section 3, “Free-energy formulations”), and was also already present in Bitbol and Luisi (2004).

### Self-organization and teleology in Kant

Weber and Varela (2002) cogently noted that Kant's *Critique of Judgment* (1790) contains a strikingly prescient vision about a self-organizational account of life. Therein, Kant claims that life cannot be derived at all from the mechanical laws of Newtonian physics, which only postulated “efficient causes” and not “end causes”^6^: nature appears to act “as if” it had purposes, and in fact the only satisfactory way to describe life involves adopting a teleological perspective. Furthermore, and here the anticipation of the concept of autopoiesis is stunning indeed, Kant reasons that differently from a human artifact (e.g., a watch), a living organism is composed by parts that not only—as in artifacts—exist for the purpose of making each other (and the whole artifact) function, but also materially produce each other: “A thing exists as a natural purpose *if it is* (though in a double sense) *both cause and effect of itself*” and also “such a product as an *organized* and *self-organizing* being can be called a *natural purpose*” [*Critique of Judgment*, as cited in Weber and Varela (2002)]. However, in the *Critique of Judgment*, this is not regarded yet as a constitutive principle: it does not say anything about how things are objectively, nor can it be presented as a scientific claim. It is only deemed a regulative principle, by which Kant means an orienting conceptual arrow to guide further scientific research.

Weber and Varela (2002) explore this Kantian premonition about the complexity of biology by showing how Kant's agnosticism about the legitimacy of teleology in scientific accounts of life (i.e., his unstable position between teleology and teleonomy, where the latter regards natural purposes simply as convenient descriptions) may be resolved within the theoretical framework of autopoiesis. The emerging identity and sense-making property of autopoietic agents are presented by the authors as an expression of immanent teleology: in a living organism “all relations of cause and effect are also relations of means and purpose” (Weber and Varela, 2002)^7^. A prefiguration of this idea, the authors argue, can be found in Kant's *Opus Postumum* (1804), where the transcendental principle was already somehow embodied as a consequence of Kant's realization that nothing but the living body can be the basis for the *a priori* categories he had postulated in the *Critique of Pure Reason*.

Our proposal to consider the transcendental as a core principle in biology does in fact partially overlap with the arguments in Weber and Varela (2002), but perhaps with a more specific focus on the biological *a priori* structures as a constitutive feature of autopoiesis. This can be seen as a biologically-rooted attempt to follow in the steps of the “Copernican revolution” advocated by Kant in the *Critique of Pure Reason*, where he argued that the properties that we can assign to the objects are nothing but the very preconditions for knowing the objects themselves and, consequently, that we cannot know the objects *per se* but only inasmuch as they fit our predetermined sensory and intellective apparatus. In our opinion, while underscoring the necessity of *a priori* structures for cognitive activity, it is also important to remark that such structures represent in fact a recapitulation of the successful coupling of organism and environment across multiple time scales (Maturana and Varela, 1980) within the organism's “flesh”. This perspective, we believe, may help establishing a more balanced base for the creative pursue of mutual constraints (Varela, 1996) or “generative passages” (Lutz, 2002) between first- and third-person data originally advocated by the neurophenomenology programme.

### The transcendental in the bacterium

As a first example of transcendental embodiment, we will focus briefly on the bacterial process of chemotaxis that has been used before to illustrate embodied cognition in its most elementary form^8^ (Varela et al., 1991; Varela, 1997; Thompson, 2007). As reviewed by Wadhams and Armitage (2004), almost all bacteria in which motility has been shown are chemotactic, i.e., their movement is biased toward regions with high concentration of nutrients or low concentration of toxic chemicals. In homogeneous environments, bacteria change direction very frequently, producing random movement. If nutritive or toxic chemicals are present in the environment, bacteria like *Escherichia Coli* change the pattern of motion of their flagella so as to bias their direction. Flagella are the most complex structure in bacterial anatomy, capable of two types of rotation. Clockwise rotation of flagella leads to tumbling, a continuous random change of direction, while counter-clockwise rotation promotes swimming, i.e., motion along a steady course. The switch from one modality to the other is possible thanks to the action of several metabolic networks, including events of protein phosphorilation leading to DNA transcription, which originate at the surface membrane. The membrane contains different chemoreceptors that, being rigidly set both anatomically and genetically before the newborn cell encounters any chemical attractor or repellant, formally predetermine the sensing. We can notice here a minimal transcendental relationship with the environment, expressed by a non-representational form of cognition, that impinges on formal structures synchronically definable as *a priori*: it is the presence of such structures that shapes the very limits of the organism's world and effectively defines its “cognitive domain.”

While chemotaxis is arguably the most intuitive and evident form of embodiment of the transcendental paradigm in the bacterial world, the same interpretative perspective could be applied to other features of bacterial biology, particularly to interindividual processes. Let us take plasmid horizontal transfer through bacterial conjugation (Willetts and Wilkins, 1984) as an example. A plasmid (Lederberg, 1952) is an extrachromosomal DNA molecule present in the bacterial cytoplasm in defined copy numbers and capable of autonomous replication. The phenotypic characters encoded by plasmids are not usually necessary for cell survival since their products are only seldom useful proteins, such as enzymes responsible for drug resistance, toxins, and molecules acting as iron-carriers known as syderophores (Crosa, 1989). Notably, the copies of a plasmid can be transferred not only to daughter cells during bacterial fission, but also *between cells*, thanks to the production of cytoplasmic hair-like connections (conjugative pili), within a process known as bacterial conjugation (Bradley, 1981); conjugative pili are in turn encoded by genes localized on special plasmids, called conjugative plasmids. From our perspective, this process is useful in illustrating a fundamental feature of transcendentality, namely its multi-layered nature. More specifically, in this case, the *a priori* structures represented by the plasmids can bridge the inter-individual gap to become invariants at the population level, thus providing the horizon for a “social” cognitive domain. In the immune function and in the nervous system, as will be shown, this aspect becomes even more evident.

### The transcendental in the immune function

Kant thought that science (Newtonian physics, in particular) was based on what he called *a priori synthetic judgments*, i.e., statements where the *a priori* mathematical element shapes an *a posteriori* experienced content (e.g., “all matter is quantitatively conserved”). The mystery of how nature accepts such an aprioristic constraint is the trigger for all of Kant's aforementioned speculations. Some intriguing parallels with these concepts can be drawn in the study of immune function. A crucial component of our immune defence from pathogens is mediated by proteins known as immunoglobulins (Ig) or antibodies. An Ig is formed by four polypeptidic chains bound together in a Y-shaped structure that can be found either embedded in the surface membrane of a specific type of lymphocytes, the B cells, or free in biological liquids (Edelman, 1991). The extremity of every chain in the upper part of the Y contains a region (*paratope*) specialized for the recognition of a complementary aminoacidic pattern (*epitope*) present in the exogenous protein (*antigen*) to be neutralized. B cells expressing surface self-reactive Igs, i.e., recognizing epitopes belonging to the organism they are meant to defend, are normally eliminated thanks to special mechanisms. These processes lead to the survival of only B cells that are able to distinguish “self” from “non-self” structures (Cornall et al., 1995).

As in the bacterium's case, structural analogues of the Kantian *a priori* determination of experience can be sought at multiple scales. From a general point of view, two aspects should be highlighted. First, the antigen-antibody complex can be seen as a biological analog of an *a priori* synthetic judgment, for it comprises an *a priori* formal component (the paratope, which already embodies in a key-lock fashion the epitope's shape that it may encounter), and an *a posteriori* content (the portion of the world that is singled out as an epitope); notably, the variability spectrum of all paratopes is almost completely predetermined by complex mechanisms of DNA recombination (Hozumi and Tonegawa, 1976). Second, such transcendental dynamics entails the emergence of a form of self or identity.

Transcendentality could also be seen at work in other immunologic phenomena. For instance, the process leading to the amplification of an antigen-specific B cell population occurring after the initial epitope recognition (in order to produce a sufficient quantity of Igs to block an infection) follows the same rules. This mechanism takes place thanks to signaling interactions between B and T cells, mediated by small molecules called cytokines (Lanzavecchia, 1990). Once a B cell recognizes a pathogen through the aforementioned paratope-epitope complementation, the whole antigen is internalized by the cell, degradated into peptides, hence complexed with specific membrane proteins, and finally presented to T cells, within the process of T cell activation. T cells, recognizing these very peptides through a receptor, produce cytokines and direct them only toward the B cells by which they have been activated. As a result, B cells both proliferate and produce more Igs against that specific pathogen (Parker, 1993). From the interpretative perspective proposed in this paper, multilayered and multifaceted transcendental interactions can be discerned within this process: the epitope conformation recognized by the Ig on the B cell membrane and the aminoacidic sequence of the peptide recognized by the T cell receptor are structurally very different, with hardly anything in their steric conformation leading back to the same pathogen. However, their linkage is achieved by means of what can be seen as a complex transcendental apparatus that goes beyond the mere *a priori* determined shapes of antibodies and manifests itself in the whole interaction between B and T cells, including Igs, T cell receptors and cytokines. This represents a hierarchically higher, but still fully embodied, instance of immunologic transcendentality. The multilayered nature of the transcendental will be further explored within the nervous system in the following section.

## 3. Transcendental neurophenomenology

The original, pragmatical aim of neurophenomenology was to establish a disciplined approach to first-person experience that would enable a mutual enlightenment of phenomenology and neuroscience by reciprocal constraints (Varela, 1996). We believe that the theoretical aspect of the neurophenomenology programme could benefit from a stronger emphasis on the transcendental properties of the brain as a biological, autopoietic system. In the following subsections, we provide concrete examples of such an interpretation, while in a subsequent section we will show how a transcendental perspective may help to shed light onto the potential usefulness of contemplative practices for neurophenomenology.

### Action-oriented perception in the mirror system as a transcendental mechanism

Let us begin by considering transcendentality in the context of sensory experience, that is, as the relationship between the *a priori* form within which subjective experience arises and the *a posteriori* content of that experience (e.g., the perceptual category of a human face *vs.* the percept of a specific face). At a first stage of approximation, when low-level sensory information from primary cortices in the brain impact on higher-level associative cortices, the latter can be seen as embodying *a priori* (albeit malleable) structures that are necessary for meaning to arise *a posteriori* when matching up with suitable input^9^. Since our aim here is not to provide an exhaustive review of all cortical processes amenable to be viewed under a transcendental perspective, we will limit our reflection to a few specific networks, beginning with the so-called “mirror neurons” within the premotor cortex of the primate [see Rizzolatti and Craighero (2004) for a review].

The importance of the sensorimotor system in cognition is exemplified by the notion of “affordance” (Gibson, 1979), a term indicating a pragmatic opportunity for motor interaction presented by an object to the subject, which cognitively qualifies the former in terms of the behavior it “affords.” The relevance of such implicit motor components for the perception of an object, regardless of the actual explicitation of movement, has been greatly enriched by the later discovery of “mirror” properties of specific neuronal populations, originally identified in the ventral premotor cortex (area F5) of the macaque monkey (di Pellegrino et al., 1992; Gallese et al., 1996; Rizzolatti et al., 1996). Neurophysiological recordings from this area showed neurons discharging both when monkeys performed a specific motion *and* when they saw another monkey or operator executing the same action. Similar properties have been reported for neurons in various human cortical areas, notably in fronto-parietal circuits, which have been proposed to encode the very goal of the action (Rizzolatti and Sinigaglia, 2010)^10^. In the mirror neuron theory, this coding represents a parsimonious mechanism for the direct understanding (i.e., without the need of inferential reasoning) of motor goals and, through a larger-scale process of integration, of the intentions of others, thus playing a crucial role in social cognition. Studies in the monkey (Sakata et al., 1995) also showed that specific neurons within the anterior intraparietal area (AIP), which has strong reciprocal connections to area F5, respond both to object grasping by the animal itself (in light as well as in darkness) and to object observation alone, supporting a Gibsonian view of perception. Although this type of response is not often reported in studies on humans, a similar phenomenon has been observed within putative human analogs of mirror areas such as the ventral premotor cortex (vPM) and intraparietal lobule (IPL): following a training where geometrical shapes were associated to hand actions, vPM and IPL showed an adaptated response from shape observation to both hand action execution and observation, additionally suggesting that mirror properties may arise as the result of simple sensorimotor coupling throughout life (Press et al., 2012).

In order to underscore the transcendental principle within the mirror system function, it is useful to draw a comparison with the previous example from immunology. In the former case, the Ig possesses *a priori* the specific morphological features corresponding to the antigen to be neutralized; in the latter case, motor patterns that predetermine and select specific features of the object (e.g., the shape of a mug handle) represent necessary *a priori* forms for its meaningful perception. Both can be seen as instances of embodiment of the transcendental, as we have thus far discussed.

### Core and historical sense of self: transcendentality and the default-mode network

Considering that experience may not necessarily concern a contingent external stimulus, but also a fully-endogenous product of mentation (e.g., the recall of episodic memories or the envisioning of future scenarios), leads us to examine the question of the self. The transcendental here appears to be at work in at least two fundamental configurations. First, as a form of prereflective self-consciousness accompanying the experience of not only external objects (Zahavi, 2002), but also of mentally-evoked scenarios, and giving it a core sense of “mineness”. Second, as the historically-determined mental content from previous experiences now acting as an *a priori* for the interpretation of novel suitable stimuli. Notably, both layers of the transcendental are operating in the course of everyday experience (although this may not be the case for altered states of consciousness and in contemplative practices). The intrinsic circularity that can be noticed in the above description, where perception is determined by structures that are in turn shaped by the active coupling of subject and environment over time, is in fact a telling sign of the enactive nature of both mind and body. This feature can be illustrated more clearly by examining the relationship between ongoing and evoked neural activity in the brain.

Already early in the history of human brain imaging it was noted that frontal cortical regions display an elevated blood flow during passive but wakeful rest (Ingvar, 1979). A later meta-analysis (Shulman et al., 1997) and a further investigation by Raichle et al. (2001) established that there is a consistent set of brain regions that show high metabolic activity at rest and decrease their activity during demanding externally-oriented tasks (Gusnard and Raichle, 2001; Gusnard et al., 2001). This set of regions, that came to be known as the “default-mode network” (DMN) (Raichle et al., 2001), includes the medial prefrontal cortex (MPFC), posterior cingulate/retrosplenial cortex (PCC/Rsp), the lateral inferior parietal lobe (IPL), and the hippocampal formation/parahippocampal gyrus (HF/pHipp) in the medial temporal lobe. While the different regions of the DMN have been traditionally associated with specific functions—e.g., the medial temporal lobe subsystem, together with PCC/Rsp, is implicated in successful retrieval of detailed information from memory (Vincent et al., 2006); the MPFC subsystem is typically engaged by self-referential judgment tasks (Gusnard et al., 2001; Qin and Northoff, 2011)—the functional and adaptive significance of the DMN as a whole remains elusive.

As several works reviewed in Buckner et al. (2008) show, one functional hypothesis is that the DMN underpins spontaneous mentation in the form of stimulus-independent thoughts (SITs), that is, thoughts detached from the contingencies of the present environment or task. The number of SITs increases during resting state compared to during demanding tasks, and indeed the activity of the DMN has been repeatedly associated with this kind of mind-wandering (Binder et al., 1999; McKiernan et al., 2003, 2006; Mason et al., 2007; Christoff et al., 2009; Smallwood et al., 2012). The activity of the DMN has also been linked to acts of “self-projection,” a general mental faculty that includes recall of autobiographical memories, theory of mind (i.e., inferring the mental content and intentions of others), future envisioning, and moral decision making (Buckner et al., 2008). All of these phenomenal correlates of the DMN share two fundamental features. First, they all entail representations of scenarios that are always alternative to the experience of the actual contingent environment; not surprisingly, some authors have proposed an adaptive explanation for this mental activity (Gilbert and Wilson, 2007) as a sort of “life simulator” for the exploration and anticipation of events and social circumstances (Buckner et al., 2008). The other feature shared by the majority of DMN-related mental processes is that the imagined perspectives are self-referenced, i.e., they are centered on the subject's self; DMN regions have in fact been shown to increase their activity when envisioning future scenarios that included the subject herself (Szpunar et al., 2007).

These experimental findings enable us to speculate that, although the activation (or the ongoing activity) of the DMN is correlatively linked to specific mental contents, it is also generally related to the sense of self. This sense of self is to be considered both as the empirical, historically-determined “I”—i.e., the one based on specific behavioral traits, interests, education and so forth—and, in a more basic sense, as the invariant character shared by all these experiential contents (as well as those elicited by external sensory stimulation), namely the noetic act of intending toward the contents themselves. Here lies in fact one of the central aims of our proposal for neurophenomenology: to identify and illuminate via the transcendental perspective the neural processes that give rise to the complexity of the noetic-noematic relationship and structure, rather than approaching the noematic or the noetic aspects separately. An important discussion of these themes can indeed be found already in Lutz (2002): the present work can be seen as extending the arguments discussed therein, with the same goal of facilitating the production of “generative passages” between first-person accounts and biological descriptions, by placing a more explicit emphasis on the transcendental structure of brain function and organization.

#### The role of the DMN in framing mental content within the autobiographical self

The extant data implicating the DMN in memory processes (both voluntary and spontaneous), suggests that it may embody a set of autobiographical *a priori* categories according to which novel mental content is apperceived and take on its peculiar and subjective meaning. As recently shown (Qin and Northoff, 2011), cortical midline structures of the DMN, in particular MPFC and PCC/Rsp, are involved in processing self-related (as compared to non self-related) stimuli (e.g., an image of a piano perceived by a pianist). While Northoff (2012) noticed indeed that the neural overlap between stimulus-dependent and resting state activity has an intriguing similarity with Kantian views of knowledge, our position is differently articulated. First, he seems to consider the historical/autobiographical sense of self as the ultimate *a priori* structure to which extrinsic stimuli should conform in order to become conscious; we find this view important but not exhaustive, within the search for the neural structure of embodied transcendentality, as will be argued in the forthcoming paragraphs. Second, Northoff's reference to Kant's legacy is treated as a heuristic to guide neuroscience, and is not really framed within an autopoietic perspective.

#### A putative role of the DMN in providing the prereflective horizon for mental content

The DMN activity has also been examined in relation to stimuli that are not explicitly related to the self. In fact, some authors have linked the elevated activity of the DMN at rest to a broad, spontaneous mode of information-harvesting in the absence of top-down focused attention (Gusnard and Raichle, 2001; Gilbert et al., 2007; Hahn et al., 2007). This functional interpretation, known as the “sentinel hypothesis” (Buckner et al., 2008), is consistent with a role of the DMN in realizing a first-person viewpoint on the world that frames and anchors all contingent percepts to the subject's identity. Notably, while the ventral portion of the PCC/Rsp node of the DMN is more involved in the retrieval and encoding of episodic memory, the more dorsal aspect is especially implicated in spatial navigation and in orienting the body in space (Vogt et al., 2006), providing a “situatedness” that may arguably be part of the “horizon” of prereflective consciousness within whose bounds mental content is staged.

#### Prereflective bodily-awareness outside of the DMN

For the sake of completeness, we should point out that prereflective self-awareness includes a sheer bodily component with affective valence (Damasio, 1999; Thompson and Varela, 2001; Legrand, 2006), whose transcendental structures may be phylogenetically very ancient (and thus more rigidly determined) and engendered by the activity of insular cortices specialized for interoception (Craig, 2002, 2009, 2011). Notably, insula and anterior cingulate, which often activate in concert, have been viewed as limbic analogs of sensory and motor cortices, respectively (Heimer and Hoesen, 2006; Craig, 2009), an interpretation that would fit nicely with a role of these regions in providing a bodily component of the sense of self related to homeostatic and basic sensorimotor loops; see Seth et al. (2011) for a recent formulation of this hypothesis in a predictive-coding framework. In a more speculative fashion, we would also like to briefly comment on how the phenomenology of the phantom limb following amputation (Ramachandran, 1998; Ramachandran and Hirstein, 1998) suggests that innate somatic *a priori* may be particularly resistant to change, a notion consistent with recent findings on its cortical correlates (Makin et al., 2013). Similarly, phantom limb pain may reflect the autopoietic attempt at survival of the neural *a priori* for limb perception, in spite of missing visual and somesthesic input, by creating an emergent nociceptive pattern that sustains the activity of the *a priori* itself via downward causation (Thompson and Varela, 2001).

### The transcendental rhythm of ongoing brain activity

In order to avoid potential misunderstandings, we would like to make clear that we are not claiming that the neural processes underpinning prereflective self-awareness exist *qua* a specific activation pattern within a single network. The entire brain is likely not to be sufficient, in fact, for this self-awareness, which may critically depend on the presence of a body (Thompson and Varela, 2001). Furthermore, since the *a priori* structures in the body and the brain are generated and shaped (both phylo- and onto-genetically) by an interaction with the environment, to limit cognition to what takes place within the cranial vault seems specious. However, the brain's contribution is crucial: its complex hierarchy of neural patterns is indispensable to orchestrate the rhythm of the transcendental process, where the intentional casting out of prereflective assumptions is followed immediately by the arising of a mental content. Such hierarchy is declined in both a spatial and a temporal dimension, as several empirical data suggest. Sadaghiani et al. (2010) review these issues arguing against a strictly segregationist view of spatial and temporal brain dynamics, i.e., interpretations of single areas or single frequencies as exclusive for certain processes. On the spatial side, networks spanning a large range of scales can be identified by functional connectivity methods, depending on how much shared temporal variance among the components of a network we decide is necessary to identify it as such. A similarly nested structure can be observed also at the temporal level, where intrinsic brain activity appears to be characterized by a 1/f spectral distribution with a predominance of very slow frequencies (Leopold et al., 2003; Nir et al., 2008). This complex-system dynamics should be studied by neurophenomenology in the light of the neural account for time consciousness proposed by Varela (1999). According to Varela's hypothesis, the diffuse structure of the “living present,” with a “pure present” at the center and a larger horizon encompassing the memory of what has just passed as well as an anticipation of the immediate future, reflects a hierarchy of neural dynamics unfolding at multiple temporal scales. In this context, phase synchrony of neural discharge is hypothesized to occur at the most basic level within the smallest sensorimotor assemblies (reflecting the “pure present”), which are in turn recruited by larger assemblies that integrate the activity of the components in a globally coherent fashion: this latter level would represent the uncompressible phenomenological granularity giving rise to the “living present” that we actually perceive. Regardless of whether this specific model is correct, it is not unreasonable to speculate that the rhythm of consciousness may directly reflect the various inertial phases in the process of transcendental recruitment of previously stored contents of experience, thus enabled as categories, for the interpretation of the present input flow. For example, it has been shown that during a decision task on Rubin's ambiguous vase-face figures, the ongoing prestimulus activity in the right fusiform face area—a region specialized for face processing—biased subjects toward the detection of a face rather than a vase (Hesselmann et al., 2008a). This finding, which has been also replicated in the domain of motion perception (Hesselmann et al., 2008b), is in agreement with the idea of a noetic self-casting of consciousness (“throwing the net out into the sea”) that we have been discussing so far.

### Free-energy formulations

This philosophical analysis of brain processes might benefit from a formalization of the transcendental relationship as the structural invariant across experience and brain processes, a feature the neurophenomenological method cannot overlook (Thompson, 2007, p. 329). In this regard, a recently proposed formulation of brain dynamics in terms of free-energy by Karl Friston appears to be especially promising (Friston and Stephan, 2007; Friston, 2009, 2010a). The free-energy principle, which builds on the early ideas of Hermann Helmholtz on perceptual processing, states that a self-organizing (autopoietic) system, in order to maintain its form and function, behaves as to minimize surprise^11^. The brain is viewed as an inferential machine following Bayesian principles that continuously brings forth predictions about the causes of sensory input and elicits actions that seek to confirm them. To this aim, the parameters specifying the internal model generating such predictions are constantly updated, thus explaining away a prediction error resulting from the discrepancy between predicted and actual input—a measure that under certain simplifying assumptions coincides with free energy. This scheme is embodied in a complex cortical hierarchy where higher level assemblies compute prediction errors and issue modification signals to the generative model, implemented at a lower level. Minimization of free-energy occurs in the brain via adjustment of three neural aspects: (i) synaptic activity, during the process of perception, (ii) synaptic gain (precision), accounting for the modulatory effect of attention on perception, and (iii) synaptic efficacy, implementing learning processes (that is, updating the model) based on experience. The free-energy principle can be seen as a dynamic system theory-based formulation of the transcendental (here fashioned in terms of Bayesian priors) as applied to brain function, a framework that we believe avoids the major pitfalls of classical reductionism and has a great potential to inspire further progress in neurophenomenology. We think this is the case because the theory, while allowing a rigorous mathematical formalization, is also sufficiently embodied to be consistent, on the one hand, with a sensorimotor-centered view of cognition and, on the other hand, with an account that sees the phenomenological transcendental as impinging on the biological one. In fact, the free-energy framework seems able to reconcile for the first time the German romantic *Naturephilosophie* and the physicalist program of Helmoltz, two divergent offsprings of Kant's original ambivalence about teleology (Weber and Varela, 2002); within this perspective, Kant's notion of the natural purpose of an organism, i.e., its tendency to conserve form and function, is recast as its incessant suppression of free-energy. Notably, the free-energy principle can apply to longer time scales as well, providing a cogent description of co-evolutionary processes that embed/embody salient features of the environment within an organism and that thus govern the formation of the *a priori* structures along the phylogenesis axis (Friston, 2010b; Friston et al., 2012).

### Neurophenomenology in clinical conditions

Finally, we would like to examine two clinical conditions—spatial unilateral neglect and schizophrenia—that, in our opinion, show the practical advantages of neurophenomenological studies conducted under the transcendental perspective. Unilateral spatial neglect is a neurological condition secondary to vascular or traumatic incidents. It is characterized by the inability to attend to and report stimuli on the side (often left) opposite to the lesion despite apparently normal vision, by an action bias toward the non-neglected hemifield, as well as by various disorders of awareness, including a denial of illness (anosognosia). The pathogenesis of neglect remains unclear despite recent promising proposals (Corbetta and Shulman, 2011). Commonly injured regions in neglect are the parietal cortex (especially IPL), the superior temporal gyrus (STG), inferior frontal gyrus (IFG) (Husain and Kennard, 1996; Karnath et al., 2001; Mort et al., 2003), as well as subcortical structures (Karnath et al., 2002; Bartolomeo et al., 2007). A recent account of neglect implicates altered connections within the right hemisphere between a dorsal fronto-parietal attention network, involved in top-down spatial attention but seldom injured in patients, and a ventral fronto-parietal attention network whose lesions have been frequently associated with non-spatial aspects of neglect (Corbetta and Shulman, 2011); this disruption of normal connectivity would in turn be responsible for altering the interhemispheric balance in the activity of the dorsal network, during both rest and task. In the light of the previous discussion about ongoing activity and its interpretation as a repertoire of priors cast out toward the world, we find this condition to be of particular interest. From a phenomenological point of view, the pathology can be interpreted as arising from an impairment of the noetic part of the transcendental movement, i.e., a failure of the basic *a priori* categories without which no perception can occur: visual input, albeit intact, remains an almost useless datum when the *a priori* it should conform to is not active anymore. We feel that such a transcendental reading of ongoing activity is not a merely speculative exercise, but could actually open up a promising avenue of research for neglect and other agnosic-like diseases.

Our second example where transcendental neurophenomenology could provide a useful interpretative framework is schizophrenia, a mental illness characterized by a dramatically altered perception of reality. So-called “positive” symptoms include auditory hallucinations, paranoid delusions and disorganized speech, while “negative” symptoms comprise affective flattening, memory and attention impairment. Preliminary data suggesting an overactive DMN in schizophrenia have recently been reported (Garrity et al., 2007; Harrison et al., 2007; Zhou et al., 2007), on the basis of the typically blurred boundary between inner mentalized scenarios and stimuli from the external environment, as well as between self and other (Buckner et al., 2008). In this case, we think that a transcendental neurophenomenology along the lines discussed so far could provide a neuroscientific counterpart to the phenomenological speculative research on psychiatric illness. With regard to the latter, it is worthwhile to mention Sass and Parnas (2003)'s interpretation of schizophrenia using Husserlian notions, where symptoms are explained as deriving from a disturbance of ipseity, explicitly defined as “myness” or prereflective self-awareness. Once again, the hypothesized link between ongoing DMN activity and prereflective self-awareness can represent a fertile ground of mutual enrichment for neuroscience and transcendental phenomenology.

## 4. Contemplative practices and neurophenomenology

Contemplative practices as found within the Buddhist and other traditions share with phenomenology a specific attention to the first-person examination of mental function in a disciplined fashion. In fact, it has been previously suggested that neurophenomenology could benefit from a closer familiarity with such practices (Varela et al., 1991; Varela, 1996), and we believe this suggestion is even more appropriate in the light of the issues discussed so far.

To this aim, we propose to look afresh at the process of meditation under the guiding notions of transcendentality and of predictive coding in the free-energy framework. A shared characteristic of many contemplative practices is the prescription to maintain a steady posture and minimize the variability of sensory input, e.g., by avoiding to change the direction of the gaze and by choosing a silent place for sitting. These instructions correspond in fact to subtracting out the adjusting manouvre of motor action and perception from the self-confirmatory loop of the transcendental process, a gesture for which we hypothesize a few important consequences: (a) the constant tension toward seeking self-confirmation in the outside world gradually abates, (b) the transcendental manifold may now begin to engage with itself instead of with external stimuli, providing partial insight into its structure to the meditator, and (c) minimization of free-energy when external stimulation is greatly reduced but attention is kept alert may prune the discrepancies among different components of the internal model, resulting in an increased consistency of the cognitive structure of the meditator, which may be at the basis of the sensation of being more “unified” often reported by contemplative practitioners. In other words, the cognitive activity *about the world* that is generated in performing adjustments to the internal probabilistic model on the basis of sensory data during everyday life, becomes cognitive activity *about the self*^12^, a perspective that affords some intriguing speculations.

First, if the core sense of an “I” exists only insofar as it keeps maximizing the evidence supporting it (by issuing appropriate actions that minimize free-energy), this incessant transcendental process at the root of cognitive activity can be also likened to a primary existential “suffering,” a lingering and mostly subconscious anxiety that such confirmation may one day fail. It is not difficult here to see the analogy with the Buddhist idea of a fundamental “craving”^13^ lying at the heart of human suffering (*duḥkha*, in Sanskrit), and with the related notion of impermanence (*anitya*, in Sanskrit) (Mizuno, 1996). Within the soteriological prospect of Buddhism, contemplative practices provide a privileged access to this state of affairs and, most fittingly with our interpretative framework, are said to be conducive to a reduction of the “mineness” or ego-centered quality of experience (Austin, 1998, 2009). More tangentially, a fascinating correspondence could be drawn between the notion that cognitive activity is inescapably endowed with a self-confirmatory nature, and the expounding of the Buddhist doctrine in Dōgen Zenji (1200–1253 CE), the founder of the Sōto school of Zen in Japan, centered on the idea that “delusion” and “enlightenment” are “ever intimate” and thus cannot in any way be considered separately (Kim, 2007).

Second, it is generally thought in contemplative traditions that sustained practice foster the capability of realizing the constructive and provisional quality of our conceptual structure, which in turn may lead to a greater freedom of choice for behavior and enhanced creativity—claims that have found some supporting evidence from recent psychological and neuroimaging research on semantic processing, mental flexibility, and creativity (Pagnoni et al., 2008; Moore and Malinowski, 2009; Colzato et al., 2012; Greenberg et al., 2012; Ostafin and Kassman, 2012). The allegedly privileged stance afforded by meditation to observe the transcendental process at work may be especially valuable for the neurophenomenological enterprise, in that it may provide specific hints for the investigation of neural processes embodying different instances and layers of the cognitive *a priori* endowment. This may be especially fruitful for what concerns the function of the DMN in enacting and looking out for environmental confirmation of a model of the self based on autobiographical memories^14^; a number of changes in the dynamics and structure of the DMN associated with meditative practices have in fact recently been reported (Pagnoni et al., 2008; Brewer et al., 2011; Taylor et al., 2011; Hasenkamp et al., 2012; Josipovic et al., 2012; Pagnoni, 2012; Taylor et al., 2013), although the phenomenological aspects of experimental design in this field have yet to be fully explored [but see Garrison et al. (2013a,b) for pioneering steps in this direction].

## 5. Summary and conclusions

In this paper we have underlined the importance of Kant's and Husserl's legacies as methodological resources for better characterizing the reading of the living world in terms of a transcendental relationship. To this aim, we first compared proto-living or minimal autopoietic systems to bacteria in order to analyze elementary biological analogs of the subject's *a priori* knowing apparatus. We then focused on the immune function for an illustration of the processes of transcendentality and identity-emergence in a system of greater complexity. We finally examined some neuroscientific data and clinical conditions under a transcendental perspective, concluding with a section on the relevance of contemplative practices for neurophenomenology.

Our main message is that a transcendental reading of brain activity can provide a useful framework where neurophenomenology may better unfold its potential. Outside from an autopoietic and enactive conception of biology, neurophenomenology loses its peculiarity, namely that of a method that conjugates organically and not by mere juxtaposition the deep analogs between mind and life. However, while in extant theoretical accounts (Cosmelli et al., 2007; Thompson, 2007) it is widely assumed that the brain as viewed by neurophenomenology is a complex self-organizing system, and important experimental work bridging neurodynamical patterns and first-person accounts has already been carried out (Lutz et al., 2002; Cosmelli et al., 2004), our feeling is that what we see as the very core of this self-organization, namely, the presence of biologically-steeped *a priori* structures, has often not been sufficiently underscored (but see Weber and Varela (2002) for a notable exception). What we hope to have provided in this paper is a suggestion about what, in our opinion, is the crucial feature that any neurophenomenology-inspired dynamic model of the brain should be centered upon, i.e., a formalization of the structure/content or noetic/noematic relationship. Such relationship, originally noticed and investigated by philosophers with regard to subjective experience, can be seen as embodied at multiple levels of life evolution. The brain has a peculiar status in this picture, given the unique complexity of its hierarchical architecture that makes it capable of embodying a very large repertoire of possible interactions, not only between organism and environment, but among different subcomponents of its own structure as well. Therefore, while also bacteria and the immune systems can be viewed as endowed with an elementary form of cognitive activity, every organism with a sufficiently large brain produces a world that is infinitely richer in meaning.

Some of the neurodynamic models reviewed by Cosmelli et al. (2007) as potentially useful for neurophenomenology include indeed a formalization of this kind, e.g., of the relationship between content and context of a given experience in terms of a structured temporal binding of thalamo-cortical activity (Llinás and Ribary, 2001), or of the subjective feeling of “expectancy” preceding novel sensory stimulation on the basis of previous experience (*protention*, in phenomenological parlance) in terms of a dynamical linkage between the activities of enthorinal cortex/hippocampus and sensory areas (Freeman, 2000). These, however, can be seen as particular instances of one fundamental principle shaping life and brain function, i.e., the transcendental, which in our opinion has not yet been granted a sufficient importance in neurophenomenology.

A critical issue with the neurophenomenological search for a correlation between “structural invariants” of experience [e.g., the temporal character of the transition from one quality of experience to another; see Cosmelli et al. (2004)] and brain dynamics, is that formal models “can only capture the structure of a domain; they cannot capture its intrinsic nature” (Bayne, 2004). But what is consciousness's intrinsic nature? It is subjectivity, a sense of “prereflective and preconceptual ‘ipseity’ ” (Cosmelli et al., 2007): in other words, transcendentality. The term transcendental is here used in its pure Husserlian notion, indicating the pre-given horizon within whose limits the subjective world discloses. Our proposal consists mainly in broadening this notion to express the fundamental character of life. Such generalization of the transcendental, similar to that of cognition in the autopoiesis framework (Varela et al., 1991), does not only extend its meaning outside the domain of phenomenology i.e., to biology) but also within it, emphasizing a hierarchical recapitulation of the transcendental process at various levels of complexity. Thus, the transcendental comes to signify any subject/object (intentional) relationship where the experience of the object is determined *a priori* by the formal structures of the subject, in a Kantian spirit. When this perspective is applied to neuroscience, it may aid the understanding of, e.g., autobiographical memory traces as *a priori* categories imbuing with meaning external stimuli relevant to the self, or neural patterns of premotor activity as *a priori* forms for the perception of “actionable” objects and situations. Once again, we would like to stress that the transcendental should be understood as multilayered and multifaceted, both on the neural and on the phenomenological side.

If we consider the body (and the brain) not only as “transcendentally lived” (Thompson, 2004) but also as transcendentally organized, it may be easier to capture in a formal model life's and phenomenology's common intrinsic nature. Notably, Thompson and Varela (2001)'s approach to the neuroscience of consciousness, notwithstanding its originality in being the first direct attempt to a neurophenomenological enterprise and in introducing a synergetics-informed account of the relationship between brain and mind, does not, in our opinion, fully address the transcendental as the core aspect of bodily processes underpinning consciousness. On the other hand, other neurodynamic models of consciousness (Engel and Singer, 2001; Engel et al., 2001; Llinás and Ribary, 2001), despite including specific aspects of embodied transcendentality, attribute them to a peculiar organization of neural processes and do not explicitly address them as radical features of life [but see Freeman (2000), for a proposal that is somewhat closer in spirit to ours].

How is such interpretation of neurophenomenology to be implemented in a contemporary disciplined programme? This is a matter for future research to investigate. We will only suggest here that such inquiry should be rooted in the notion that the transcendental process occurs at multiple levels, and thus select the levels to focus on according to the study's specific aim. At one end of the spectrum, neurophenomenology's goal of bridging the mind-body problem may be facilitated by addressing transcendentality at its most basic level, that is, the fundamental noetic act of prereflective awareness. Such attempt may usefully investigate intrinsic, ongoing brain activity as an autopoietic, prediction-casting process, that infuses meaning to both the internal and the external world. To this end, regularities in the relationship between phenomenological and neural transcendental features should be established, possibly capitalising on the enhanced introspective skills of subjects acquainted with contemplative practices. At a different level, neurophenomenology may explore its practical applicability to the study of neurological and psychiatric conditions (e.g., agnosia, neglect, memory impairments, schizophrenia). In this context, a putative disruption of the transcendental process, both in its phenomenological and neural aspects, should be investigated as a potential avenue for novel therapy developments.

### Conflict of interest statement

The authors declare that the research was conducted in the absence of any commercial or financial relationships that could be construed as a potential conflict of interest.

## Footnotes

^1^In philosophy, the notions of *a priori* and *a posteriori* assume the sensory, empirical experience as the reference point. For instance, the formal statements of logic are considered generally *a priori*, because they do not need validation from, or are somewhat independent of sensory experience; on the other hand, a specific aroma is generally considered an *a posteriori* object as it relies completely on sensory experience.

^2^In the Preface to the second edition of the *Critique of Pure Reason*, Kant writes: “If the intuition must conform to the nature of the objects, I do not see how we can know anything of them *a priori*. If, on the other hand, the object conforms to the nature of our faculty of intuition, I can then easily conceive the possibility of such an *a priori* knowledge. Now, as I cannot rest in the mere intuitions, but if they are to become cognitions […], here again there are two courses open to me. Either, first, I may assume that the conceptions […] conform to the object—and in this case I am reduced to the same perplexity as before; or secondly, I may assume that the objects, or, which is the same thing, that experience […] conform to my conceptions—and then I am at no loss how to proceed.” (Kant, 1990).

^3^A chemical reaction is said to be autocatalytic if at least one of the reaction's products is a catalyst for the reaction itself.

^4^Micelles are spheroidal organic structures, typically made of hydrophobic lipid molecules known as surfactants, capable of self-generation and self-assembly.

^5^We use the term “organ” here in the original sense of the greek word *òrganon*, i.e., “instrument” or “means”.

^6^In our practical life, Kant explains, these two orders of causes are circularly linked by the exclusive role of intelligence: a house I possess may be the efficient cause of the money I gain from renting it, but the idea of that gain represents in turn the end cause of the construction of the house in the first place.

^7^See Nagel (2012), for a recent articulation of the legitimacy of the teleological principle *vis-à-vis* the mechanistic paradigm.

^8^The use of bacteria to exemplify the concept of autopoiesis is also particularly fitting given that the overwhelming majority of biomass on Earth is bacterial.

^9^The approximation here concerns the fact that there is likely a transcendental component in the lower-level neuronal circuits as well. The transcendental mechanism may in fact be at work down to the minimal levels of neural assemblies exhibiting self-organizing properties.

^10^This encoding is realized across different scales, from neurons specific for one single observed and executed act, to others specific for a broader goal achievable by different motor strategies.

^11^Surprise is formally defined as the improbability of sampled sensory data under a generative model of the agent's world. The fact that surprise is conditional on a model is essential to explain explorative behavior and thus to avoid the “dark-room” paradox (Friston et al., 2012).

^12^Cognitive activity is intended here in its more general, autopoietic acception, which includes processes occurring below the level of consciousness.

^13^Where craving can be construed as the emotion-laden, intentional aspect of self-confirmation.

^14^The putative role of the DMN in bringing forth autobiographical *a priori* categories that affect experience echoes suggestively the Buddhist notion of *karma*, indicating “seeds” of past action that bound the possibilities of current behavior.
